# Hierarchical Porous Carbon Derived from Sichuan Pepper for High-Performance Symmetric Supercapacitor with Decent Rate Capability and Cycling Stability

**DOI:** 10.3390/nano9040553

**Published:** 2019-04-04

**Authors:** Hengshuo Zhang, Wei Xiao, Wenjie Zhou, Shanyong Chen, Yanhua Zhang

**Affiliations:** 1 Research Institute for New Materials Technology, Chongqing University of Arts and Sciences, Yongchuan, Chongqing 402160, China; zhs19941108@163.com (H.Z.); wenjie_zhou@aliyun.com (W.Z.); jluchensy@163.com (S.C.); 2 Faculty of Materials and Energy, Southwest University, Chongqing 400715, China

**Keywords:** Sichuan pepper, porous carbon, symmetric supercapacitor, biomass, KOH activation

## Abstract

Hierarchical micro-mesoporous carbon (denoted as HPC-2 in this study) was synthesized by pre-carbonization of biomass Sichuan pepper followed by KOH activation. It possessed well-developed porosity with the specific surface area of 1823.1 m^2^ g^−1^ and pore volume of 0.906 cm^3^ g^−1^, and exhibited impressive supercapacitive behaviors. For example, the largest specific capacitance of HPC-2 was tested to be ca. 171 F g^−1^ in a three-electrode setup with outstanding rate capability and stable electrochemical property, whose capacitance retention was near 100% after cycling at rather a high current density of 40 A g^−1^ for up to 10,000 cycles. Furthermore, a two-electrode symmetric supercapacitor cell of HPC-2//HPC-2 was constructed, which delivered the maximum specific capacitance and energy density of ca. 30 F g^−1^ and 4.2 Wh kg^−1^, respectively, had prominent rate performance and cycling stability with negligible capacitance decay after repetitive charge/discharge at a high current density of 10 A g^−1^ for over 10,000 cycles. Such electrochemical properties of HPC-2 in both three- and two-electrode systems are superior or comparable to those of a great number of porous biomass carbon reported previously, hence making it a promising candidate for the development of high-performance energy storage devices.

## 1. Introduction

Due to the increasing deterioration of environment and the urgent demand for clean and renewable energy, advanced energy storage technologies have gained considerable attention [1,2,3]. Among diverse energy storage systems, electrochemical capacitor, so-called supercapacitor, is recognized as one of the most promising next-generation candidates because of its ultrahigh power density, short charge/discharge time, long cycle life, stable performance, and broad working temperature, which has found many applications in consumer electronics, backup power supply, hybrid electrical vehicle, and implantable medical devices [3,4,5]. Based on the charge storage mechanism, supercapacitors can be classified as electrical double-layer capacitors (EDLCs) and pseudocapacitors [6,7]. The former relies on the electrostatic accumulation of electrolyte ions at the electrode/electrolyte interface to store energy, while the later achieves this point by the reversible Faradaic redox reactions occurring near the electrode surface [6,7]. Overall, compared with pseudocapacitors, EDLCs offer higher power density but lower energy density, meanwhile, they are more environmentally friendly and much safer [7].

Over the past two decades, a wide range of compounds have been developed as electrode materials for supercapacitors, such as conductive polymers, metal oxides, sulfides, and hydroxides [6,7,8]. Although these pseudocapacitor electrode materials exhibit high specific capacitance, their low electrical conductivity, environmental harmfulness and poor cycling stability still severely restrict their commercialization. As a consequence, more and more interest is focused on carbonaceous materials owing to their good electrical conductivity, excellent stability, and low cost. Various carbonaceous materials like carbon nanotubes, graphene, carbon aerogel and porous carbon have been frequently reported for energy storage devices [9,10]. Especially, porous carbon derived from natural substances stands out and becomes a hotspot in the field of supercapacitor, since its renewable precursor, abundant resources and easy fabrication process make it quite suitable for large-scale production and application [11,12,13,14,15,16,17]. For instance, some effective strategies, including one-step activation, template method as well as combination of carbonization and activation, have been proposed to synthesize porous carbon by adopting wheat straws, rice bran, almond shells, pig nails, plant leaves, corn, silk and starch as raw materials [5,11,12,13,14,15,16,17]. These biomass-derived porous carbon products feature substantial micropores (˂2 nm), resulting in large specific surface area and presenting high specific capacitance. Unfortunately, their rate performance and cycling stability are often far from satisfaction due to lack of sufficient mesopores (2–50 nm) and macropores (>50 nm), which favor fast transport, penetration, and diffusion of electrolyte ions within electrodes [4,7]. To upgrade the capacitive performances of biomass carbon, construction of hierarchical porous architecture with interconnected micro, meso, and macropores is regarded as an ideal design [4,7]. Nevertheless, it remains a challenge to choose appropriate biomass and approach to synthesize hierarchical porous carbon with outstanding electrochemical properties for high-performance supercapacitors.

**Figure 1:**
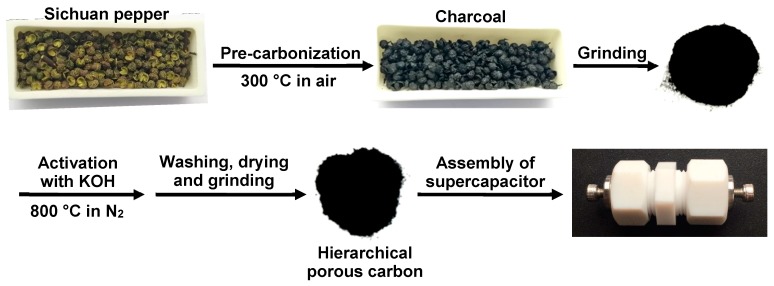
Schematic representation of the preparation of hierarchical porous biomass carbon derived from Sichuan pepper.

Sichuan pepper, widely known as Huajiao in China, denotes the fruits of *Zanthoxylum* in the plant family Rutaceare, which is a popular spice for culinary purposes and can be used as drug in traditional Chinese medicine for anti-microbial, anti-inflammation and analgesia [18,19]. Moreover, plenty of extracts are able to be obtained from Sichuan pepper as well, and they are extensively added as food ingredients in many savory goods and beverages [18,20]. Despite these merits and efforts, it is of great significance to expand the application of Sichuan pepper into other important and valuable aspects such as energy storage area. In the present work, this raw material was employed as a sustainable and green precursor to prepare hierarchical porous carbon via pre-carbonization followed by KOH activation under high temperature (Figure 1). Thanks to the interconnected micro-mesoporous structure, high specific surface area, and well-developed porosity, the currently synthesized porous biomass carbon displayed satisfactory capacitive performances with the maximum specific capacitance of ca. 171 F g^−1^ in a three-electrode system, the highest energy density of 4.2 Wh kg^−1^ in a symmetric supercapacitor cell, as well as outstanding rate capability and cycling stability. The current method to fabricate hierarchical porous carbon is straightforward and suitable for mass production. Consequently, a type of cost-effective and environmental friendly carbon electrode materials is developed for energy storage, while providing a new promising application of Sichuan pepper.

## 2. Experimental

### 2.1. Chemicals

Sichuan pepper was bought from local supermarket. Potassium hydroxide (KOH), hydrochloric acid (HCl), polyvinylidene fluoride (PVDF), *N*-methyl-2-pyrrolidone (NMP), nickel foam and acetylene black were purchased from Sinopharm Chemical Reagent Co., Ltd. (Shanghai, China). All the reagents were used without further purification. Deionized water was employed throughout this study.

### 2.2. Fabrication of Sichuan Pepper-Derived Hierarchical Porous Carbon

6 g of Sichuan pepper was collected in a corundum boat and then put into a muffle furnace for pre-carbonization at 300 °C (ramp rate: 4 °C min^−1^) for 2 h in air. Afterwards, a part of the resulting charcoal (0.6 g) was ground to powder, infiltrated with 1 mL of ethanol, and mixed with 1 mL of aqueous solution of KOH to form a slurry, which was sufficiently dried at 110 °C for 12 h in an electric oven. For further activation and carbonization, the resulting mixture was transferred into another corundum boat and heated in a quartz tube furnace at 800 °C (ramp rate: 2 °C min^−1^) for 2 h in N_2_ atmosphere. At last, the rude product was successively washed with 1 M HCl and abundant deionized water, followed by drying and grinding, thus yielding hierarchical porous carbon. To optimize the porosity of hierarchical porous carbon, the mass ratio of KOH to the intermediate charcoal during the above synthetic process was set as 0:1, 1:1, 2:1, and 3:1, and the corresponding final products were denoted as HPC-0, HPC-1, HPC-2, and HPC-3 for convenience, respectively.

### 2.3. Characterizations

Field emission scanning electron microscopy (FESEM) inspections were performed on a Zeiss GeminiSEM 300 scanning electron microscope working at an acceleration voltage of 3 kV. Transmission electron microscopy (TEM) examinations were observed on a Tecnai G2 F20 transmission electron microscope operating at an acceleration voltage of 200 kV. Powder X-ray diffraction (XRD) patterns were measured on a Tongda TD-3500 diffractometer adopting Cu Kα (λ = 0.154156 nm) as the radiation source. Raman analysis was carried out on a Zolix Finder One Raman spectrometer, and its incident light is green laser with the wavelength of 532 nm. N_2_ adsorption/desorption experiments were done on a Micromeritics ASAP 2020 analyzer at the temperature of liquid nitrogen (77 K). The specific surface area (S_BET_) of the samples was estimated according to the Brunauer‒Emmett‒Teller (BET) theory, their micropore surface area (S_mic_) was analyzed by the t-plot method, and their mesopore surface area (S_meso_) was obtained by subtracting S_mic_ from S_BET_. The total pore volume (V_t_) was calculated based on the amount adsorbed at the relative pressure of P/P_0_ = 0.99, the micropore volume (V_mic_) was deduced utilizing Dubinin–Radushkevich analysis in the relative pressure range from 10^−4^ to 10^−2^, and the mesopore volume (V_meso_) was obtained by subtracting V_t_ from V_mic_. The pore size distribution was determined by the original density functional theory, which was combined with non-negative regularization and medium smoothing.

### 2.4. Electrochemical Tests

The fabrication of working electrodes was briefly described as follows. Firstly, the active material (i.e., the currently synthesized carbon materials), acetylene black and PVDF binder were accurately weighed with the mass ratio of 75:15:10 and mixed together in an agate mortar. Then, appropriate amount of solvent NMP was introduced, and the resulting mixture was mildly ground to generate a homogeneous slurry. Finally, the as-prepared black slurry was brushed onto one side of rectangular nickel foam (1 cm × 3 cm in size, which was employed in three-electrode setup) or circular nickel foam (1.1 cm in diameter, which was used for assembly of supercapacitor cells) with the coated area ~1 cm^2^, followed by pressing under the pressure of 2 MPa and drying in a vacuum oven to give several electrodes. The amount of active material loaded on each working electrode was 2.1 ± 0.4 mg.

All the electrochemical measurements were taken on a CHI 760 E electrochemical workstation (Shanghai Chenhua Instrument Co. Ltd., Shanghai, China). The electrochemical investigation of three-electrode system was conducted in aqueous electrolyte of 2 M KOH, where the rectangular nickel foam loaded with carbon materials, Hg/HgO electrode and platinum foil were used as the working, reference and counter electrodes, respectively. Moreover, To build a symmetric supercapacitor cell, two pieces of circular nickel foam coated with the same amount of hierarchical porous carbon (i.e., HPC-2) were wetted by aqueous solution of 2 M KOH, paired face to face with a polypropylene separator sandwiched in between, and then sealed in a two-electrode device. The capacitive performances of the resulting supercapacitor cell were systematically surveyed in such two-electrode configuration. Cyclic voltammetry (CV) tests at varied scan rates were done from −0.9 to 0.1 V and from 0 to 1 V in three-electrode and two-electrode systems, respectively. Likewise, galvanostatic charge/discharge (GCD) curves at different current densities were recorded in the potential range from −0.9 to 0.1 V and from 0 to 1 V in three-electrode and two-electrode systems, respectively. Electrochemical impedance spectroscopy (EIS) was determined in the frequency range from 100 kHz to 0.01 Hz at open circuit, and the signal amplitude of this measurement was 5 mV.

## 3. Results and Discussion

### 3.1. Materials Characterization

**Figure 2:**
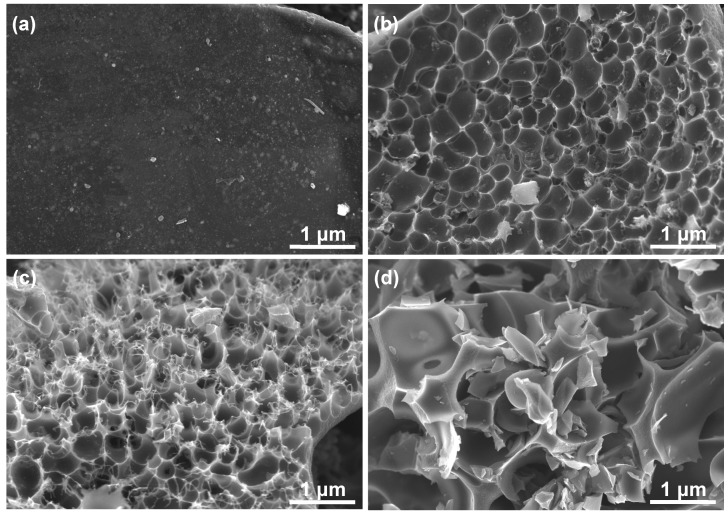
FESEM images of (**a**) HPC-0, (**b**) HPC-1, (**c**) HPC-2 and (**d**) HPC-3, which were synthesized by adjusting the mass ratio of KOH to the intermediate charcoal as 0:1, 1:1, 2:1, and 3:1, respectively.

**Figure 3:**
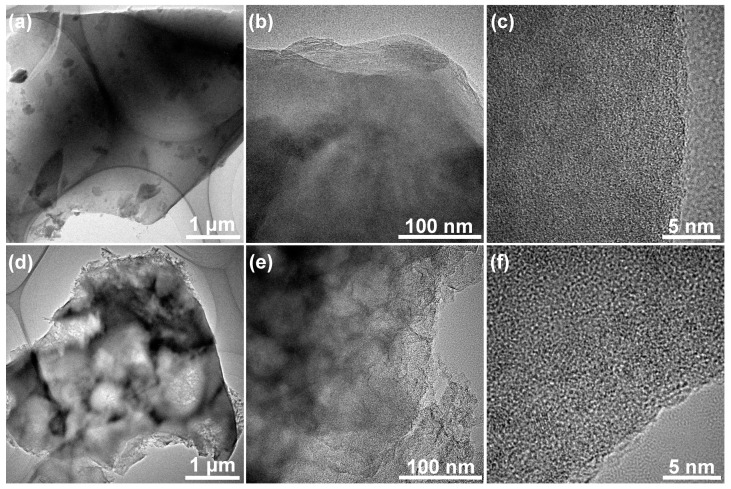
TEM images of (**a**–**c**) HPC-0 and (**d**–**f**) HPC-2 at different magnifications.

Figure 2 displays the microscopic morphologies of the products synthesized in this work. As can be clearly seen, HPC-0, which was prepared by direct carbonization in the absence of KOH, seems to be solid, nonporous and relatively smooth (Figure 2a), and such structure is not beneficial for electrolyte ion diffusion during charge/discharge processes. After activation with a certain amount of KOH, a large number of pores are created, and honeycomb-like cellular framework with thin pore walls can be found in the resulting carbon materials of HPC-1 and HPC-2 (Figure 2b,c). The porosity of the HPC-2 is more developed than that of HPC-1 due to deeper etching arising from more dosage of KOH. However, excessive KOH brings about the damage of interconnected porous nanostructure in the material HPC-3 as presented in Figure 2d. Figure 3 is a group of TEM images of HPC-0 and HPC-2. The former keeps the intrinsic noncrystalline structure and has no porous texture (Figure 3a–c), while hierarchical porous architecture (Figure 3d–f) and numerous micropores (Figure 3f) are visible in the later. Based on the FESEM and TEM examinations, it can be concluded that the feeding amount of KOH plays an essential role in creating three-dimensional porous network, and the optimum weight ratio of KOH to charcoal is 1:2, which gives rise to well-developed porosity of carbon material HPC-2.

**Figure 4:**
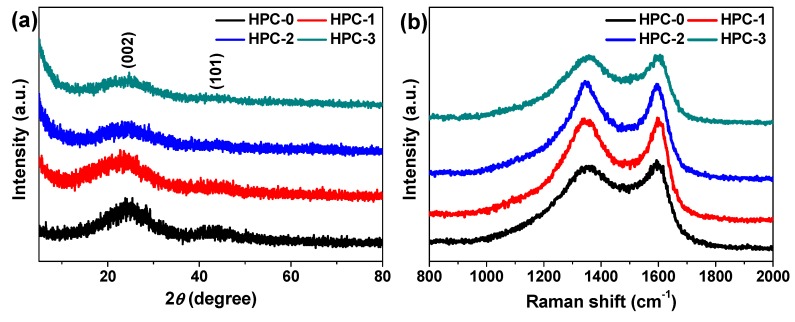
(**a**) Powder XRD patterns and (**b**) Raman spectra of HPC-0, HPC-1, HPC-2, and HPC-3.

Figure 4a exhibits the powder XRD patterns of HPC-0, HPC-1, HPC-2, and HPC-3. All of them show two typical peaks at around 2θ = 25° and 44°, which can be well indexed to the (002) and (101) planes of hexagonal graphite (JCPDS no. 41-1487) [14,17,21], respectively, suggesting that the diffractions should originate from graphitic carbon [14,17,21]. The intensity of the peak located at 2θ = 44° gradually fades out as elevating the dosage of KOH, demonstrating the increasing extent of disorder [14,21]. The Raman spectra of the four specimens are given in Figure 4b, where a pair of characteristic peaks can be noticed at ~1350 and ~1595 cm^−1^, corresponding to the D and G bands of carbon matters, respectively [14,17,21,22]. The D band is related to the structural defects and disorders, while the G band stands for the sp^2^-hybridized graphitic carbon structure [14,17,21]. The intensity ratio of D to G band (I_D_/I_G_) is usually used to evaluate the graphitization level of carbon materials, namely, the lower value of I_D_/I_G_, the higher degree of graphitization [14,17,21]. The value of I_D_/I_G_ is determined to be 1.0, 1.03, and 0.99 for HPC-1, HPC-2, and HPC-3, respectively, and all of which is higher than that of HPC-0 (0.94). Such result suggests that KOH activation indeed causes textural defects and disorders on carbon materials due to the generation of enormous nanopores, which also coincides with the outcome of XRD characterizations [14,21,23].

**Figure 5:**
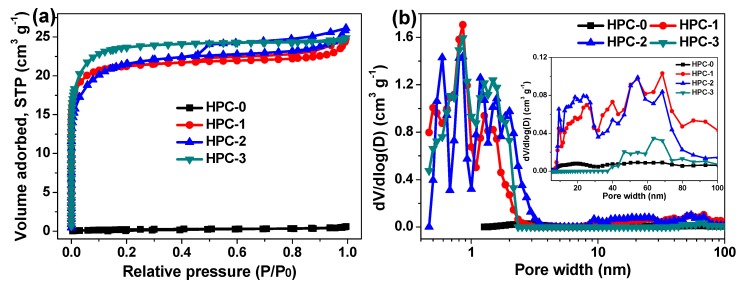
(**a**) N_2_ adsorption/desorption isotherm curves and (**b**) Pore size distribution diagrams of HPC-0, HPC-1, HPC-2 and HPC-3.

The porous nature of the currently synthesized carbon materials were further characterized by N_2_ adsorption/desorption measurements, and the corresponding isotherm curves are exhibited in Figure 5a. As envisioned, HPC-0 gives an almost horizontal line, suggesting its nonporous structure [14,21]. For HPC-1 and HPC-2, combined isotherm curves of type I and IV are obtained, showing steep uptake of nitrogen at low relative pressure (P/P_0_ ˂ 0.1) and typical hysteresis loops at relative pressure of 0.5–1.0, which are indicative of the existence of plentiful micropores and mesopores in the two samples [3,4,14]. Unlike HPC-1 and HPC-2, hysteresis loop is absent in the isotherm curve of HPC-3, which can thus be classified into type I isotherm, that is, HPC-3 is rich in micropores but lack of mesopores [4,21]. Such porous characteristics are also intuitively revealed by their pore size distribution diagrams as depicted in Figure 5b and the inset. The porosity parameters of these specimens are presented in Table 1. It is found that the specific surface area and total pore volume of HPC-0 are only 20.2 m^2^ g^−1^ and 0.021 cm^3^ g^−1^, respectively, while those of HPC-1, HPC-2, and HPC-3 are close to each other, which reach up to ~1900 m^2^ g^−1^ and ~0.9 cm^3^ g^−1^, respectively. Even so, the mesopore surface area (681.5 m^2^ g^−1^) and mesopore volume (0.459 cm^3^ g^−1^) of HPC-2 are far more than those of HPC-1 and HPC-3, once again testifying its superior hierarchical porous texture and well-developed porosity. The high specific surface area, large total pore volume as well as hierarchical porous architecture with reasonable distribution of micropores and mesopores would promote the contact area between electrode material (i.e., HPC-2) and electrolyte, facilitate the diffusion of electrolyte ions, and offer rich active sites as locations for charge accumulation, and hence, greatly contribute to the capacitive performances of HPC-2 electrode and the corresponding supercapacitor device [4,14,21].

### 3.2. Electrochemical Evaluations

**Figure 6:**
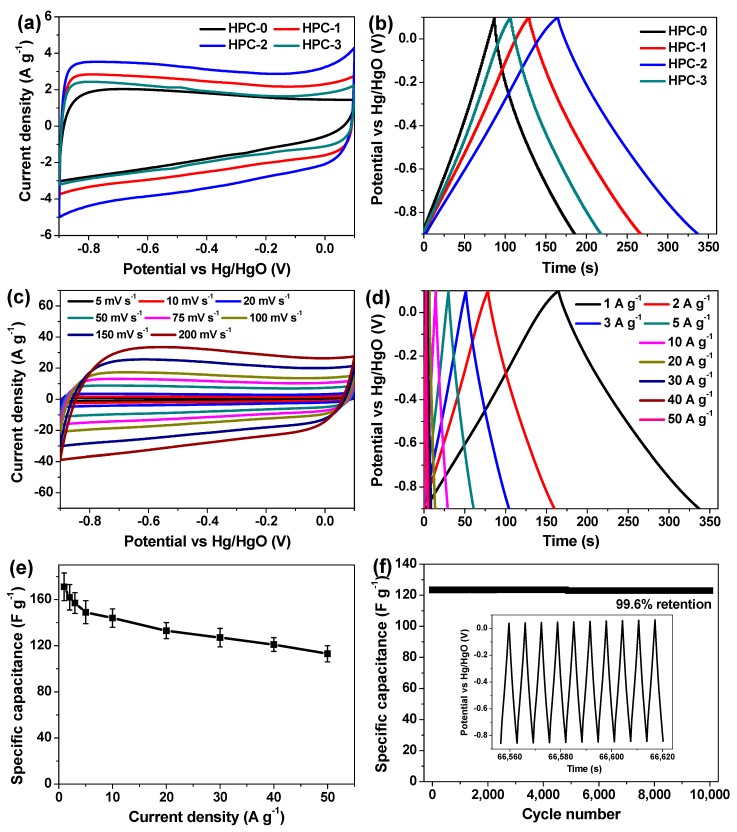
Electrochemical characterizations of the currently fabricated carbon electrodes measured in a three-electrode system. (**a**) CV curves of HPC-0, HPC-1, HPC-2, and HPC-3 electrodes acquired at the identical scan rate of 20 mV s^−1^; (**b**) GCD curves of HPC-0, HPC-1, HPC-2, and HPC-3 electrodes obtained at the same current density of 1 A g^−1^; (**c**) CV curves of HPC-2 electrode tested at varied sweep rates; (**d**) GCD curves of HPC-2 electrode tested at different current densities; (**e**) the relationship of specific capacitance of HPC-2 electrode versus current density; and (**f**) the cyclic performance of HPC-2 electrode for continuous charge/discharge at rather a high current density of 40 A g^−1^ for up to 10,000 cycles; the inset shows the charge/discharge curve of such cycling test for the final 10 cycles.

The electrochemical properties of the currently synthesized carbon materials were first studied in 2 M KOH electrolyte by using a three-electrode setup. Figure 6a depicts their CV curves at the scan rate of 20 mV s^−1^. A seriously distorted rectangular CV curve is obtained for HPC-0. Unlike HPC-0, HPC-1, HPC-2 and HPC-3 show typical quasi-rectangular shapes, demonstrating the EDLC characteristics as supercapacitor electrode materials for charge storage [4,14,21]. Notably, the area enclosed by the CV curve of HPC-2 is significantly larger than that of the others, manifesting the enhanced capacitance of the sample [9,14]. Such result is further verified by their GCD tests at the same current density of 1 A g^−1^ (Figure 6b), since the discharge time of HPC-2 is the longest [9,14]. The better electrochemical behaviors of HPC-2 possibly benefit from its larger mesopore surface area and mesopore volume to a great extent, which could offer broad channels for shortening the electrolyte ion diffusion distance during charge/discharge processes [14,21]. Figure 6c presents the CV curves of HPC-2 at different scan rates, and the rectangular-like shape is maintained even swept at 200 mV s^−1^, reflecting its excellent rate performances [21]. Figure 6d is a group of GCD curves of HPC-2 measured at current densities from 1 to 50 A g^−1^. The discharge curves are almost linear and mirror-symmetric to the charge counterparts with very small IR drop, indicating the good electrical conductivity, desirable charge/discharge activity, and high reversibility of such electrode [4,8,14]. The specific capacitance of this electrode can be deduced according to the following Equation (1),

C_m_ = It/ΔVm (1)

where C_m_, I, t, ΔV, and m represent the specific capacitance of the tested working electrode (F g^−1^), the discharge current (A), the discharge time (s), the potential change during a complete discharge process (V), and the mass of active material coated on the working electrode (g), respectively [14,15,16]. As a result, the C_m_ value of HPC-2 is calculated to be 171 ± 12, 162 ± 11, 157 ± 9, 149 ± 10, 144 ± 8, 133 ± 7, 127 ± 8, 121 ± 6, and 113 ± 7 F g^−1^ at the current densities of 1, 2, 3, 5, 10, 20, 30, 40, and 50 A g^−1^, respectively. Its C_m_ alteration as a function of current density is profiled in Figure 6e as well. Although the specific capacitance gradually decreases as the current density goes up, the C_m_ value acquired at the current densities of 10 and 50 A g^−1^ remains as high as 84.2% and 66.1% of the initial one (i.e., the C_m_ value acquired at the current density of 1 A g^−1^), respectively, once again demonstrating the prominent rate capability of HPC-2 electrode. To investigate its cyclic performance, repetitive GCD measurement was performed at the current density of 40 A g^−1^ for up to 10,000 cycles. Surprisingly, there is almost no capacitance decay from beginning to end, and the capacitance retention of HPC-2 electrode reaches up to 99.6% after the entire test (Figure 6f). In addition, the final 10-cycle charge/discharge curve still retains the quasi-isosceles triangular shape (inset of Figure 6f), commendably illustrating the exceptional long-term cycling stability of the HPC-2 electrode.

Previously, a great number of porous biomass carbons have been synthesized and utilized as electrode materials for supercapacitor applications [21,22,23,24,25,26,27,28,29,30,31,32]. On the whole, when evaluated in three-electrode systems, lots of porous biomass carbon electrodes deliver the highest specific capacitance lower than 160 F g^−1^ [24,25,26,27,28,29,30,31], and their rate capability is relatively poor as well, because, compared with their maximum specific capacitance, more than 16% drop is usually inevitable at the current densities not exceeding 10 A g^−1^ [11,14,16,21,23,32,33,34,35]. Also, an evident loss of capacitance (often over 10% decay as compared to the initial capacitance) is observed for a variety of porous biomass carbon electrodes after continuous charge/discharge (commonly less than 10,000 cycles) at the current densities lower than 10 A g^−1^ [3,7,21,23,33]. By contrast, our currently developed HPC-2 electrode releases the highest specific capacitance of ca. 171 F g^−1^ at the current density of 1 A g^−1^, features outstanding rate capability with the specific capacitance at the current density of 10 A g^−1^ still as high as 84.2% of the maximum one, and fulfills excellent cyclic performance with inappreciable capacitance fading after cycling at rather a high current density of 40 A g^−1^ for up to 10,000 cycles. It is assumed that the interconnected hierarchical micro-mesoporous architecture, unique morphology, high specific surface area and large pore volume of HPC-2 should be responsible for its superior capacitive properties, which provide extensive transport channels and abundant active sites for the adsorption/desorption of electrolyte ions during charge/discharge processes [14,21,32].

**Figure 7:**
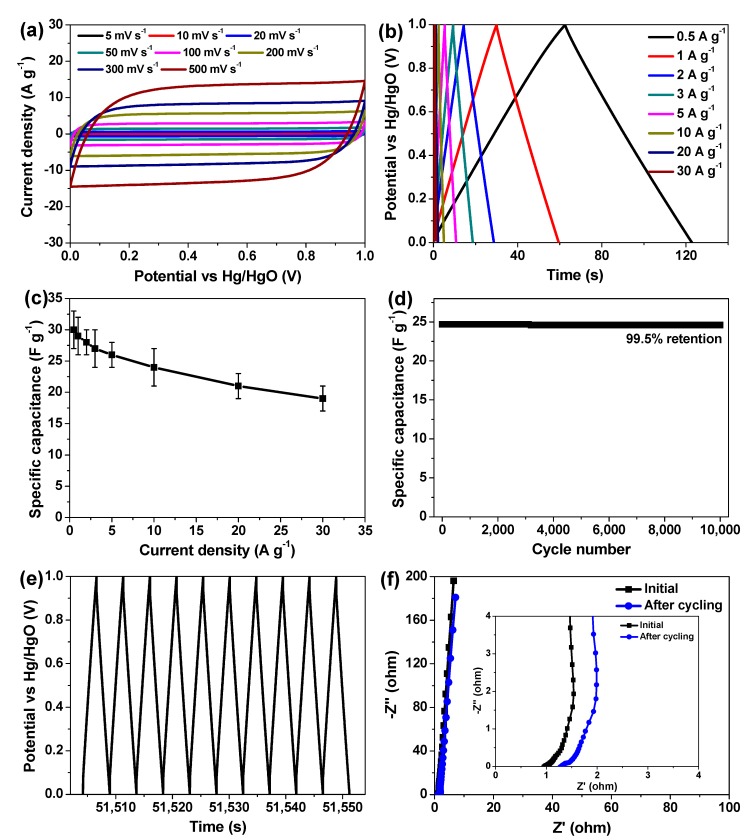
Supercapacitive behaviors of single symmetric supercapacitor cell of HPC-2//HPC-2. (**a**) Its CV curves measured at a set of sweep rates; (**b**) its GCD curves obtained at a series of current densities; (**c**) its specific capacitance as a function of current density; (**d**) its cyclic performance for repetitive charge/discharge at a high current density of 10 A g^−1^ for over 10,000 cycles; (**e**) its last 10-cycle charge/discharge curve of such cycling stability test; and (**f**) its initial Nyquist plot (black curve) and the one after such cycling stability test (blue curve); the inset is the zoom view of the Nyquist plots for high frequencies.

To reveal the practical supercapacitive device behaviors, a symmetric supercapacitor cell (i.e., HPC-2//HPC-2) was built by using the currently developed HPC-2 as electrode material and 2 M aqueous solution of KOH as electrolyte. Its detailed electrochemical characterizations were conducted and the results are shown in Figure 7. Clearly, all of the CV curves, even the one scanned at a very high speed of 500 mV s^−1^, are close to rectangular shapes with no redox peak and dramatic distortion (Figure 7a), testifying to the expected EDLC charge storage mechanism, ideal capacitive behaviors, as well as rapid charge/discharge property [9,14,21,36]. The GCD curves of such supercapacitor cell at different current densities are presented in Figure 7b, showing isosceles triangular shape with decent mirror-image symmetry and small potential drop, which are indicative of the low internal resistance and non-Faradaic supercapacitive characteristic [9,14,21]. In terms of Equation (1) and these GCD curves, the specific capacitance of the cell (C_cell_) is deduced to be 30 ± 3, 29 ± 3, 28 ± 2, 27 ± 3, 26 ± 2, 24 ± 3, 21 ± 2, and 19 ± 2 F g^−1^ based on the total weight of active material (~4.2 mg) within one supercapacitor device at the current densities of 0.5, 1, 2, 3, 5, 10, 20, and 30 A g^−1^, respectively (Figure 7c). Noticeably, the C_cell_ value still retains 80% and 63.3% by boosting the current density from 0.5 to 10 and 30 A g^−1^, respectively, implying the remarkable rate capability of HPC-2//HPC-2 cell, which outperforms that of many existing porous biomass carbon-based aqueous supercapacitors [4,5,12,16], whose output potential difference is also 1.0 V. The cycling stability of the cell was examined by consecutive GCD test at a high current density of 10 A g^−1^ for over 10,000 cycles. It is found that little capacitance degradation is observed during the continuous charge/discharge process, and 99.5% of the initial capacitance is reserved after the whole measurement (Figure 7d). Besides, its GCD curve for the last 10 cycles remains good enough (Figure 7e), and its Nyquist plots before and after the cycling test give similar shape with inconspicuous difference (Figure 7f). All these findings and results convincingly confirm the excellent cyclic performance of HPC-2//HPC-2 supercapacitor cell.

The energy and powder densities of the currently developed symmetric supercapacitor cell are calculated based on the following two equations and its GCD curves shown in Figure 7b,

E = (C_cell_ΔV^2^)/7.2 (2)

**Figure 8:**
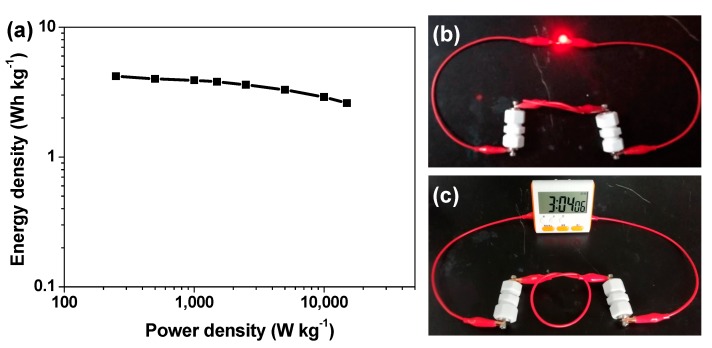
(**a**) Ragone plot of an as-assembled HPC-2//HPC-2 supercapacitor cell; (**b**,**c**) Digital photographs of a red light-emitting diode bulb and a portable timer well powered by two HPC-2//HPC-2 supercapacitor cells connected in series, respectively.

P = 3600 E/t(3)

where E, P, C_cell_ ΔV, and t stand for the energy density (Wh kg^−1^), power density (W kg^−1^), specific capacitance of the supercapacitor cell (F g^−1^), its output potential difference (V), and discharge time (s) [4,6,14]. Consequently, the HPC-2//HPC-2 supercapacitor cell offers energy densities of 4.2, 4.0, 3.9, 3.8, 3.6, 3.3, 2.9, and 2.6 Wh kg^−1^ at power densities of 250, 500, 1000, 1500, 2500, 5000, 10,000, and 15,000 W kg^−1^, respectively. To intuitively visualize these data, they are drawn in a Ragone plot (Figure 8a). Impressively, the maximum energy density (4.2 Wh kg^−1^) realized by the HPC-2//HPC-2 cell is comparable to or even better than that of a variety of existing porous biomass carbon-based symmetric supercapacitors and commercial carbon-based supercapacitors [14,24,25,30,37,38,39], demonstrating that the synthesized HPC-2 is indeed a promising carbon electrode material for development of high-performance supercapacitors. Finally, we connected two HPC-2//HPC-2 supercapacitor cells to try to power some commercial electronic products. For instance, a red light-emitting diode bulb with the rated voltage of 1.9 V and a portable timer with the rated voltage of 1.5 V were successfully driven by such tandem device after it was fully charged (Figure 8b,c), thus demonstrating its actual feasibility and usefulness as a power source.

## 4. Conclusions

In summary, employing Sichuan pepper as raw material, hierarchical micro-mesoporous biomass carbon (i.e., HPC-2) was fabricated by its pre-carbonization followed by KOH activation, which possessed interconnected cellular network, well-developed porosity, high specific surface of 1823.1 m^2^ g^−1^ and large pore volume of 0.906 cm^3^ g^−1^. When used in a three-electrode system, HPC-2 released the highest specific capacitance of ca. 171 F g^−1^ at the current density of 1 A g^−1^, exhibited desirable rate capability with the specific capacitance still as large as ca. 113 F g^−1^ at rather a high current density of 50 A g^−1^, and exhibited good cycling stability with negligible capacitance decay after consecutive charge/discharge at a high current density of 40 A g^−1^ for over 10,000 cycles. Such electrochemical properties of HPC-2 electrode are superior to those of many reported porous biomass carbon-based electrodes. As a continuation of this research, a symmetric supercapacitor cell of HPC-2//HPC-2 was further established, which delivered the maximum specific capacitance of ca. 30 F g^−1^, had outstanding rate capability and stable electrochemical behavior with capacitance retention near 100% after cycling at a high current density of 10 A g^−1^ for up to 10,000 cycles, and gave the highest energy density of 4.2 Wh kg^−1^ at a power density of 250 W kg^−1^. Such capacitive performances are comparable or preferable to those of lots of existing porous biomass carbon-based supercapacitors as well. Accordingly, it is anticipated that the currently developed hierarchical porous carbon HPC-2 may be utilized as an advanced carbon material for energy storage in practical applications.

## Figures and Tables

**Table 1:** Porosity parameters of the currently synthesized carbon materials.

Sample	S_BET_ (m^2^ g^−1^)	S_mic_ (m^2^ g^−1^)	S_meso_ (m^2^ g^−1^)	V_t_ (cm^3^ g^−1^)	V_mic_ (cm^3^ g^−1^)	V_meso_ (cm^3^ g^−1^)
HPC-0	20.2	17.3	2.9	0.021	0.008	0.013
HPC-1	1880.8	1841.4	39.4	0.858	0.73	0.128
HPC-2	1823.1	1141.6	681.5	0.906	0.447	0.459
HPC-3	2065.1	2015.1	50	0.863	0.803	0.06
